# Profile of ocular trauma in patients presenting to the department of ophthalmology at Hawassa University: Retrospective study

**DOI:** 10.1371/journal.pone.0213893

**Published:** 2019-03-28

**Authors:** Kindie Desta Alem, Demoze Delelegn Arega, Samson Tesfaye Weldegiorgis, Bekalu Getahun Agaje, Emebet Girma Tigneh

**Affiliations:** 1 Department of Ophthalmology and Optometry, College of Medicine and Health Sciences, Hawassa University, Hawassa, Ethiopia; 2 Orbis International-Ethiopia, Addis Ababa, Ethiopia; University of Debrecen, Faculty of Medicine, HUNGARY

## Abstract

**Purpose:**

To assess the pattern, presentation and risk factors of ocular trauma among patients treated at Hawassa University, Referral Hospital, Ophthalmology Department, South Ethiopia, 2014

**Methods:**

The medical records of all patients treated for ocular trauma at Hawassa University, Referral Hospital, Ophthalmology Department, during the period January 01, 2012 to July 31, 2014, were retrospectively reviewed. Trained optometrists, ophthalmic nurses and ophthalmic officer collected the data using a pretested data abstraction form. The descriptive and analytic statistics was carried out.

**Results:**

A total of 773 patients (549 males and 224 females) were included in the study. Closed globe injury was more common in males (P = 0.019) and open globe injury was significantly common in children (P < 0.001). Corneal tear was the most frequently observed finding (39.33%). Surgery, secondary to trauma, was common in open globe injury (P < 0.001). About 98% of the patients presented after 6 hours post trauma. Among 84.61% patients whose visual acuity (VA) was recorded at presentation, 12.23% had VA of 6/6 and 65.90% had VA of <3/60.

**Conclusion:**

Ocular trauma was more common in males and children. Majority of patients (98%) presented after 6 hours post trauma. Cornea was the most commonly affected ocular structure by trauma.

## Introduction

The eye represents only 0.27% of the total body surface area and 4% of the facial area, but it is the third most common organ affected by trauma after hands and feet [1].

Ocular trauma is a significant public health problem and preventable cause of visual morbidity [1–5]. It is common in developing countries and may lead to permanent visual impairment [1, 2, 4, 6, 7]. It may occur at any age in either sex [1, 4], especially among pediatric and elderly population [1]. Both hospital and population based studies indicate a large preponderance of traumas affecting males [1, 2, 4, 7–9].

According to estimates of world health organization (WHO), the global annual incidence of ocular trauma is around 55 million [3, 6, 10, 11] and worldwide blindness in 1.6 million people is due to ocular trauma [1, 7, 11–12].

Corneal tear, sclera tear and lens damage are the most frequently observed morbidities of ocular trauma [1, 2, 11, 13, 14] followed by lid and canalicular laceration, uveal prolapse, anterior chamber (AC) abnormality, retinal detachment and optic nerve avulsion [2, 11, 15, 16]. Majority of the patients were presented in to eye health facilities after 24 hours from time of trauma [1, 2, 3, 5, 6, 13, 17]. Patients reported within 24 hours of eye injury showed better visual outcome as compared to later than 24 hours presentation [2].

Socio-economic burden of ocular trauma is high involving a huge cost in human unhappiness, economic inefficiency and monetary loss [5, 6]. Its direct and indirect costs are known to run into millions of dollars annually [1].

Most (67.6%) of the patients reported for the treatment of trauma on the third day and later in a study conducted in Grarbet, Ethiopia [5]; however, no studies had been carried out on pattern, presentation and risk factors of ocular trauma in the study area. So, in view of public health importance, this study will provide information on pattern, presentation and risk factors of ocular trauma at Hawassa University, Referral Hospital, Ophthalmology Department and this will serve as the basis for planning and implementing preventive and curative measures to be undertaken by respective stack holders.

## Materials and methods

Hospital based retrospective chart review was conducted from October 25, 2014 to November 25, 2014 in Hawassa University, Referral Hospital, Ophthalmology Department. Hawassa University, Referral Hospital is found in Hawassa town, South Ethiopia, about 275Kms from Addis Ababa, the capital city of Ethiopia. The tertiary eye care center was established in 2005 by the support of Orbis International-Ethiopia. This tertiary eye care center serves approximately 16 million people of a catchment area. With pediatrics, retina and refraction clinics comprehensive eye care services are being provided in the center, both in outpatient and inpatient departments. There are currently 3 ophthalmologists, 7 optometrists, 1 ophthalmic officer, 1 cataract surgeon, 8 ophthalmic nurses and 3 primary eye care workers providing the service.

All ocular trauma cases seen in Hawassa University, Referral Hospital, Ophthalmology Department from January 01, 2012 to July 31, 2014 were identified from logbook and studied retrospectively. According to the extracted medical record numbers, from logbook, charts of patients were identified from chart room and those that fulfilled the inclusion criteria were included in to the study. Charts that lacked two or more information on the pattern, presentation or/and risk factors were excluded from the study.

The dependent variable was ocular trauma and independent variables were socio-demographic data (sex, age, residence (rural/ urban), occupation), the place where trauma occurred, type of object that causes trauma, occasion of trauma, affected eye, type of injury, affected ocular structure, secondary surgery, type of surgery, time of presentation, time of intervention and visual acuity (VA) (at presentation and after treatment). Variables were identified based on the literature and data abstraction form was developed accordingly. The form was tested in 5% of the sample (39 charts) and modified according to the available variables on charts. Post trauma ocular injury was classified based on internationally accepted Birmingham Eye Trauma Terminology System (BETTS) [18].

There were five optometrists, four ophthalmic nurses and one ophthalmic officer for data collection and one ophthalmologist for supervision. Data collectors drew all important information from the chart. The collected data was checked out for completeness, accuracy and clarity by principal investigator and supervisor on daily basis and amendments were done at the spot. Data clean up, double entry and cross-checking was done before analysis.

Data was entered and analyzed using statistical package for social sciences (SPSS) version 20. The descriptive statistics, bivariate and multivariate logistic regressions were carried out. The variables that were found being significant (p ≤0.2) in bivariate logistic regression were entered into multivariate logistic regression model. A P-value <0.05 was considered statistically significant.

Before conducting the study, ethical clearance was obtained from the Institutional Review Board (IRB) of Hawassa University, College of Medicine and Health Sciences and the IRB waived the requirement for informed consent. Official permission from the hospital was obtained. Confidentiality of the information was maintained thoroughly by excluding names as identification in data abstraction form and keeping their privacy during data collection. Charts were returned to chart room soon after collecting the necessary data. No one had access to the non-coded data except investigators, data collectors and supervisor due to responsibilities associated with the study.

## Results

### Socio-demographic characteristics

**Fig 1:**
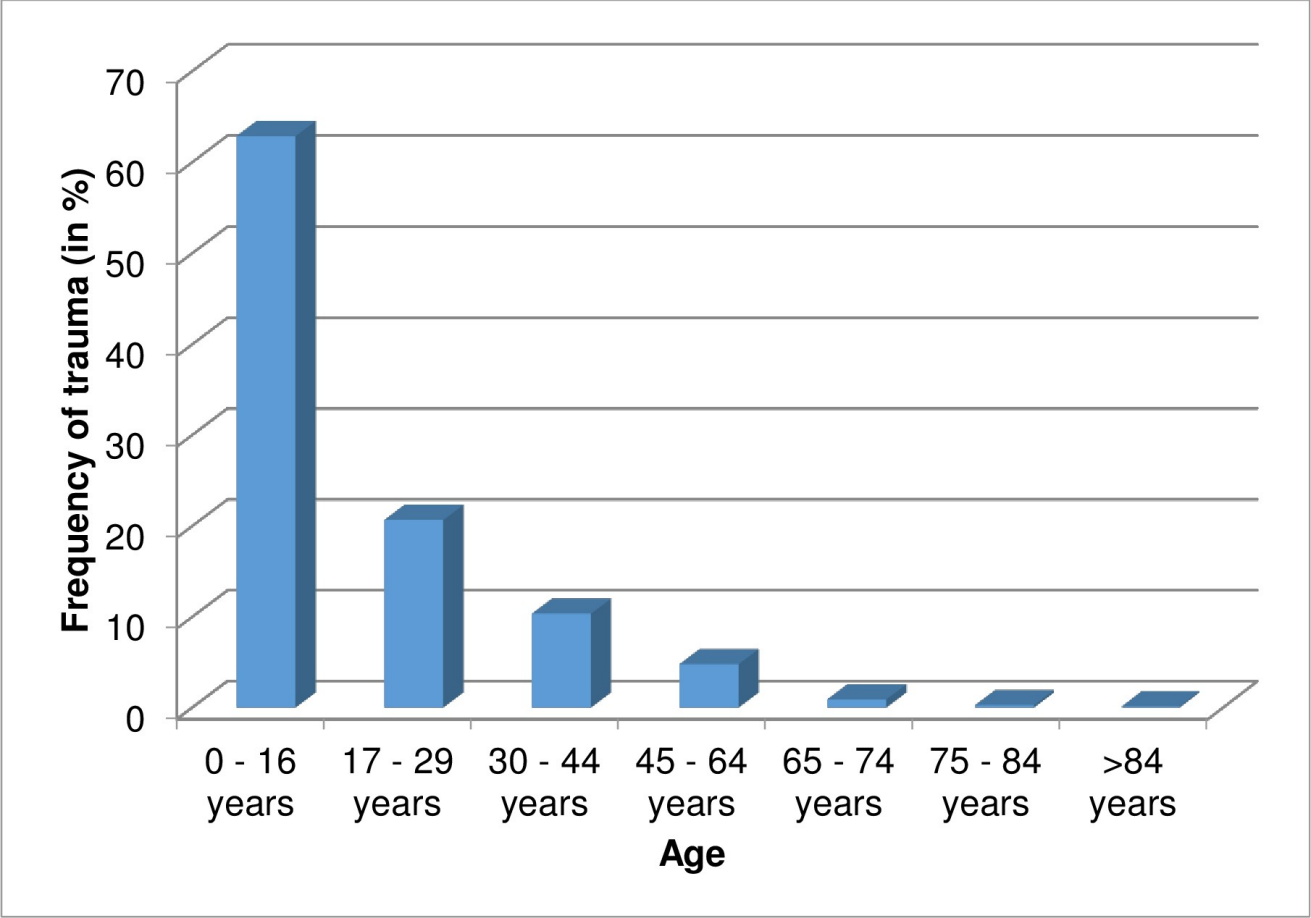
Age of ocular trauma patients at Hawassa University, Referral Hospital, from January 01, 2012 to July 31, 2014 (N = 773).

A total of 773 ocular trauma charts were reviewed retrospectively and among these 71.02% (549) were males and 28.98% (224) were females (male to female ratio–2.5:1). Patients from 2 months to 92 years old were included in the study. About 63% of ocular trauma was occurred in children (0–16 years old) and the occurrence of ocular trauma was relatively low as age increases (Fig 1).

Among patient charts where residence is recorded, the ocular trauma was frequently observed in rural residents—58.34% (451) as compared to urban dwellers—35.96% (278). Nine (1.16%) students, 2 (0.26%) farmers, 2 (0.26%) factory employees as well as 2 (0.26%) government employees were victims of ocular trauma, but the occupation was not recorded in 752 (97.28%) charts.

### Observations

According to the record review, 44.4% (343) patients right eye, 53.8% (416) patients left eye and 1.8% (14) patients both eyes were affected by trauma. Closed and open globe injuries occurred equally (47.7% and 47.1%, respectively) (Table 1).

**Table 1:** Types of ocular injury in patients treated at Hawassa University, Referral Hospital, from January 01, 2012 to July 31, 2014 (N = 773).

Type of injury		Frequency (%)
**Mechanical injury**	Open globe injury • Rupture • Laceration ✓ Penetrating ✓ IOFB ✓ Perforating	364(47.09)
		16(2.07)
		348(45.02)
		262(33.89)
		31(4.01)
		55(7.12)
	Closed globe injury • Contusion • Lamellar laceration	369(47.74)
		298(38.55)
		71(9.18)
	Adnexal	37(4.79)
**Chemical injury**		3(0.39)

IOFB, intra ocular foreign body

Maleness was associated with closed globe injury (P-value = 0.019, adjusted odds ratio (AOR) = 1.58) and open globe injury was found being significant on children (P < 0.001, AOR = 2.06). Almost in all types of ocular injury, the distribution was found being higher on children (Table 2).

**Table 2:** Correlation between the type of ocular injury and age group of patients treated at Hawassa University, Referral Hospital, from January 01, 2012 to July 31, 2014 (N = 773).

		Age of a patient				Total
		0–16	17–29	30–44	≥45	
**Type of injury**	OPEN GLOBE					
	• Penetrating	216 (83.4%)	24 (9.3%)	11 (4.2%)	8 (3.1%)	259 (100.0%)
	• IOFB	8 (26.7%)	15 (50.0%)	5 (16.7%)	2 (6.7%)	30 (100.0%)
	• Perforating	36 (65.5%)	11 (20.0%)	3 (5.5%)	5 (9.1%)	55 (100.0%)
	• Rupture	11 (73.3%)	3 (20.0%)	1 (6.7%)	0 (0.0%)	15 (100.0%)
	CLOSED GLOBE					
	• Contusion	93 (48.7%)	46 (24.1%)	32 (16.8%)	20 (10.5%)	191 (100.0%)
	• Lamellar laceration	23 (56.1%)	10 (24.4%)	6 (14.6%)	2 (4.9%)	41 (100.0%)
	ADNEXAL	21 (56.8%)	9 (24.3%)	5 (13.5%)	2 (5.4%)	37 (100.0%)
	CHEMICAL	1 (33.3%)	2 (66.7%)	0 (0.0%)	0 (0.0%)	3 (100.0%)
**Total**	486 (62.9%)	160 (20.7%)	80 (10.3%)	47 (6.1%)	773 (100.0%)

IOFB, intra ocular foreign body

Trauma has affected all ocular structures, from anterior to posterior segment. Among all (773) cases, corneal tear was the most frequently observed one (39.33%, number (n) = 304), followed by lens damage (24.45%, n = 189) (Table 3).

**Table 3:** Patterns of ocular trauma and their distribution among patients treated at Hawassa University, Referral Hospital, from January 01, 2012 to July 31, 2014 (N = 773).

S. No.	Pattern of ocular trauma	Frequency (%)
**1**	Corneal tear	304(39.33)
**2**	Lens damage	189(24.45)
**3**	Uveal prolapse	160(20.70)
**4**	AC abnormality	142(18.37)
**5**	Eyelid damage	97(12.55)
**6**	Corneal FB	65(8.41)
**7**	Sub-conjunctival hemorrhage	55(7.12)
**8**	Scleral tear	29(3.75)
**9**	Vitreous abnormality	28(3.62)
**10**	Corneal opacity	23(2.98)
**11**	Retinal detachment	8(1.03)
**12**	Others	78(10.09)

AC, anterior chamber; FB, foreign body

In most of the patients, more than two ocular structures were affected by trauma. Out of 773 patients, in 26 of them; corneal tear, anterior chamber abnormality and uveal prolapse was noticed and in 39 of them; corneal tear, anterior chamber abnormality, uveal prolapse and lens damage was observed.

### Conditions at presentation

**Fig 2:**
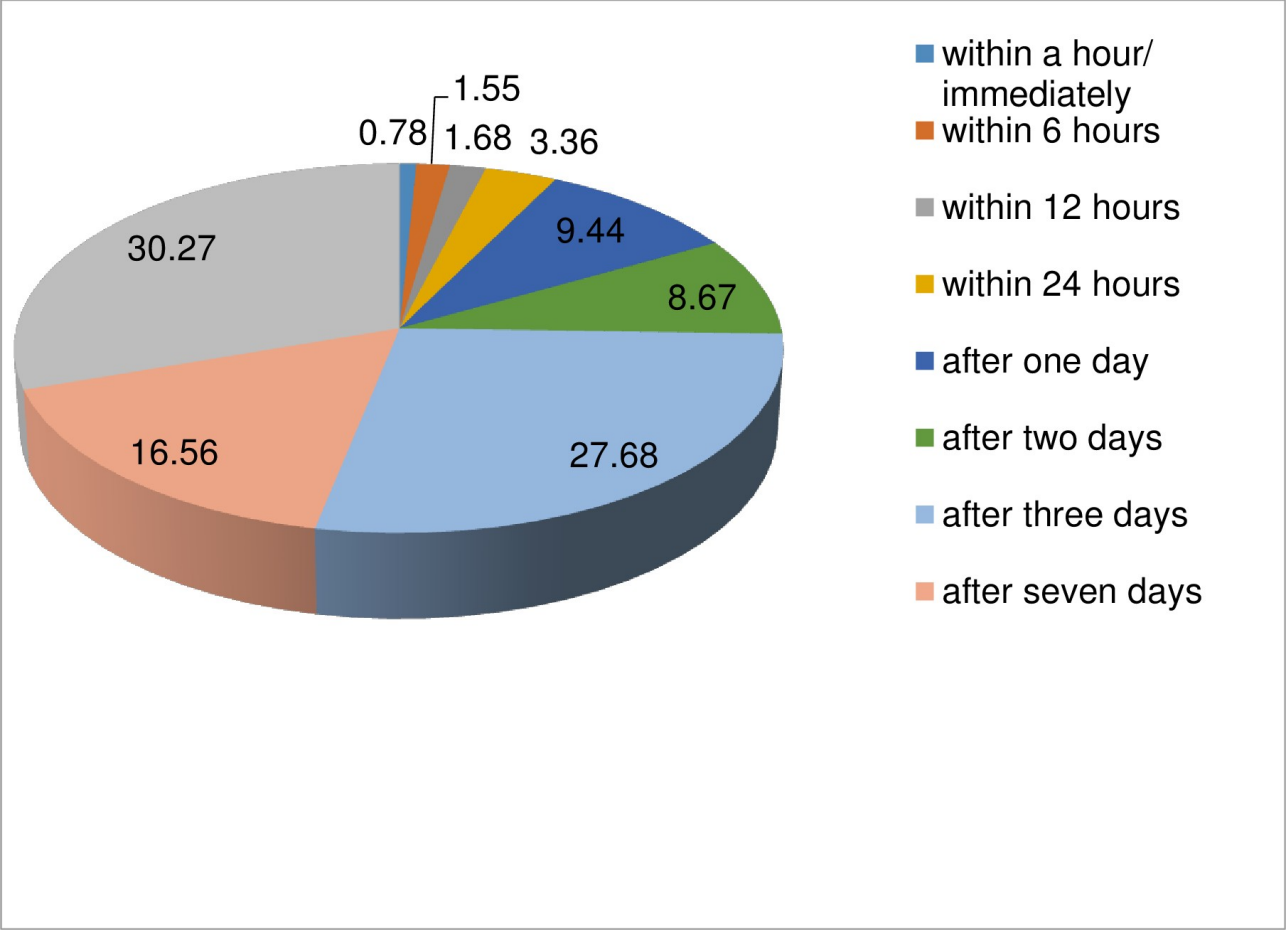
Presentation time of patients to Hawassa University, Referral Hospital, from January 01, 2012 to July 31, 2014 (N = 773).

Most of the patients (74.51%, n = 576) who encountered ocular trauma were presented to Hawassa University, tertiary eye care center after three days from trauma (Fig 2).

Majority of patients (97.02%) who presented for ocular trauma were intervened after 12 hours from the time of trauma. Only 1.29% of the total patients were treated within 6 hours. But there was no a variable associated (P<0.05) with the time of presentation.

**Fig 3:**
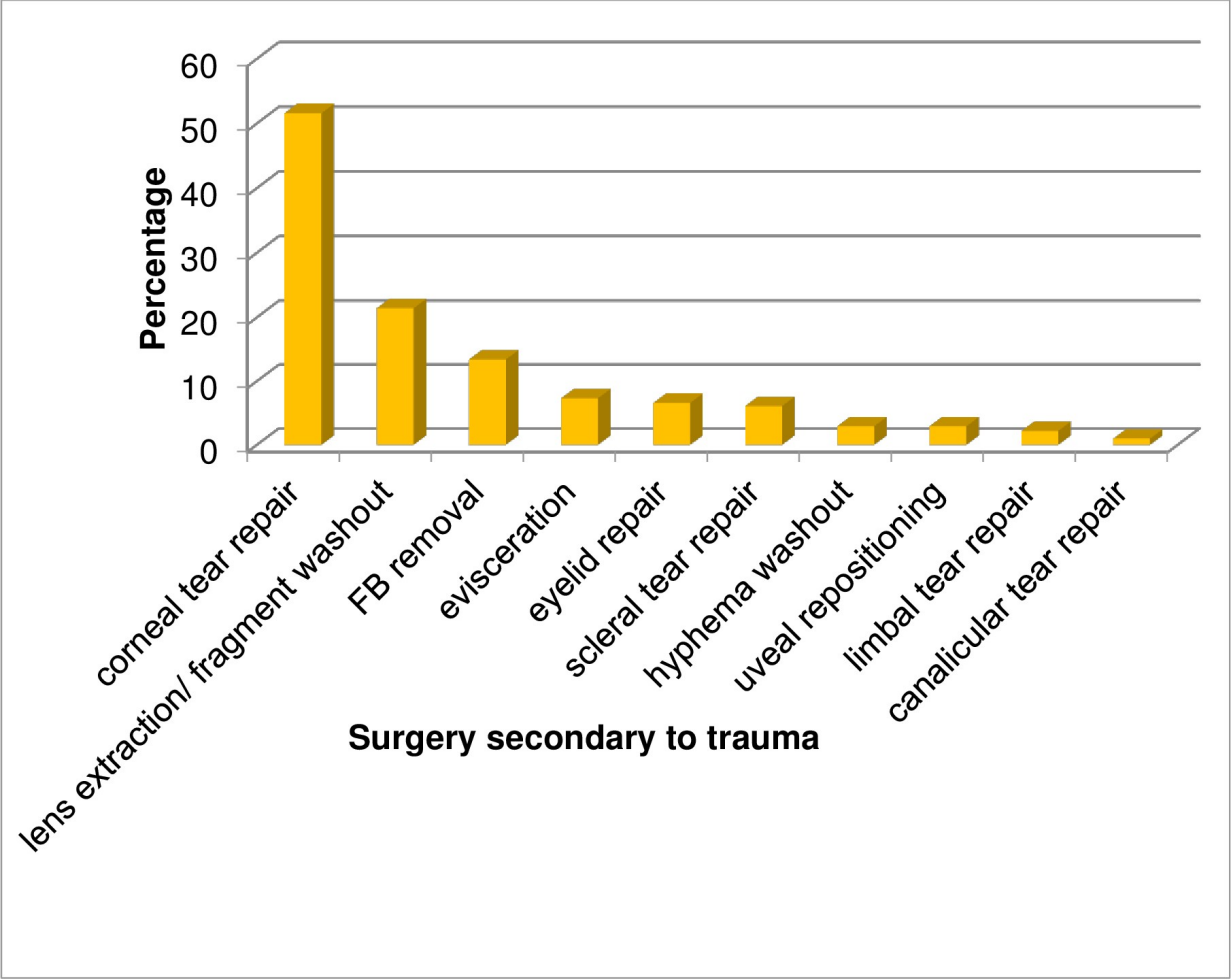
Post ocular trauma surgery of patients at Hawassa University, Referral Hospital, from January 01, 2012 to July 31, 2014 (N = 773).

Ocular surgery, secondary to trauma, was performed in 53.17% (411) patients and corneal tear repair was the most frequently performed procedure (51.8%), followed by crystalline lens extraction/ lens fragment washout (21.41%) (Fig 3).

Surgery, secondary to trauma, has shown the association with open globe injury (P < 0.001, AOR = 9.37).

Presentation VA was recorded in 83.1% (642) patients (292 right eyes, 338 left eyes and 12 both eyes). Among these, the VA was 6/6 only for 12.0% (77) patients and it was under blindness category (VA<3/60) for 62.1% (399) patients (Table 4).

**Table 4:** VA at presentation of patients treated at Hawassa University, Referral Hospital, from January 01, 2012 to July 31, 2014 (N = 773).

VA category	Frequency	Percentage
**6/6**	77	12.0
**6/9-6/18**	105	16.4
**6/24-6/48**	22	3.4
**6/60-3/60**	12	1.9
**<3/60**	399	62.1
**LF**	27	4.2
**Total**	642	100.0

VA, visual acuity; LF, light follow

### VA after treatment

**Fig 4:**
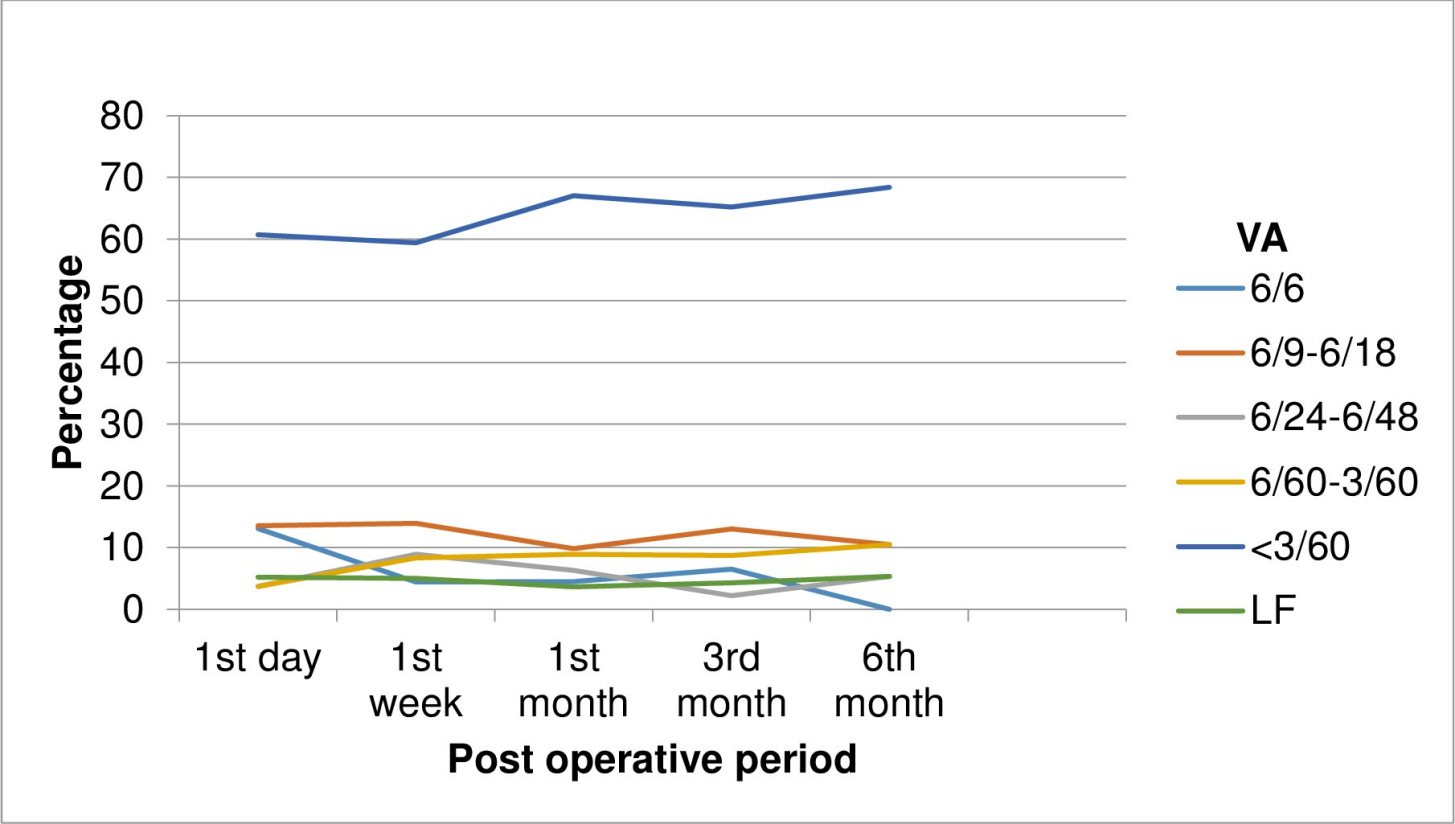
Post treatment VA of ocular traumatic patients at Hawassa University, Referral Hospital, from January 01, 2012 to July 31, 2014. VA, visual acuity; LF, light follow.

Among patients whose VA was recorded at first day after treatment, the VA was 6/6 only for 13.1% (35) patients and it was under blindness category (VA<3/60) for 60.7% (162) patients. Only small number of patients attended sixth month post trauma follow up and no patient was with 6/6 VA (Fig 4).

## Discussion

The result showed that the occurrence of ocular trauma was high in males (71.0%) and this is supported by other studies conducted around the globe; in Pakistan males accounted 80% and 75% in Peshawar and Lahore regions, respectively and in Nepal it was 69.3% and 72.3% in western Nepal and Dhulikhal hospital, respectively. Male to female ratio was 1.93:1 in India, Uttarakhand and 3:1 in Ethiopia, Grarbet. Also in Addis Ababa, Ethiopia, 74% of ocular injury was occurred in males. [3, 5, 8, 10–13]. This might be due to the reason that males are mainly engaged in outdoor activities where trauma is highly encountered.

Among patients presented for ocular trauma, children (62.87%) followed by adolescents (20.67%) accounted more than 80% and this is in agreement with other studies; Pakistan, <30 years old– 69% (Peshawar), 18–29 years old– 31% (Lahore); Kashmir valley, 16–26 years old– 75% [9, 11, 13, 16]. This might be associated with inadequacy of knowledge towards perception of accidents.

In this study, open and closed globe injuries were almost proportional, 47.07% and 47.74%, respectively. Open globe injury is almost in agreement with other studies conducted in different countries; Uttarakhand, India (45.5%) and Peshawar, Pakistan (46.18%) [6, 13]. In this study, penetrating trauma was the highest finding among open globe injuries (33.89%) and this finding was reported as 12.1% in Nigeria [1]. Also corneal tear was the most frequently observed case (39.33%) and which is much higher as compared to a study done in western India (15.2%) [2]. This will be due to the variations of the objects that cause ocular injuries.

In this study, 12.55% eyelid damage was observed, whereas this figure was higher in other studies; western India (15.66%) and Pakistan (64%) [2, 11]. AC abnormality was 18.37% in this study and this finding was higher in Pakistan; 84% (Jinnah) and >50% (Peshawar) [11, 13]. In contrast, the findings were lower in Nigeria (5.9%) and western India (8.29%) [1, 2]. Uveal prolapse was 20.70% in this study, but it was 10% of all cases in Jinnah, Pakistan [11]. About 24% lens damage was observed in this study and the higher as well as the lower values were noticed in other studies [2, 11, 13]. Vitreous abnormality (3.62%) and retinal detachment (1.03%) were among the posterior segment findings of this study and similar findings were observed also in other studies; vitreous abnormality–5.06% (western India), retinal detachment–3.68% (western India) and vitreous hemorrhage with retinal detachment–2.5% (Egypt) [2, 15]. These discrepancies might be due to differences in causes of trauma.

In this study, only 0.78% patients were presented to the hospital within a hour/ immediately, but in a study of Lahore, Pakistan, 23% of patients reported within one hour after ocular trauma [11]. In our study, only 3.36% patients were presented within 24 hours. In a study of Nigeria and Pakistan (Lahore) similar numbers of patients (38%) were presented for ocular trauma within 24 hours and also in Uttarakhand, India 52.27% patients were reported within 24 hours [1, 3, 11]. From 1^st^ to 7^th^ days, 45.79% patients reported for ocular trauma in this study, but in Nigeria 32.5% patients and in India (Uttarakhand) 12.7% patients had reported in the same period [1, 6]. After 7 days, 46.9% patients presented for ocular trauma in our study and in the same period; 6%, 12.7%, 29.5% and 63.61% patients had reported in Egypt, India, Nigeria and Pakistan respectively [1, 6, 13, 15]. The variations in presentation time might be due to the differences in distance from eye care center, poverty, awareness and transportation system across the countries.

From 86.61% patients whose presentation VA was recorded, only 12.23% were with VA of 6/6 and 65.90% were under blindness category (VA<3/60). Similarly in a study conducted in Uttarakhand, India, 56.5% patients were presented with VA of <3/60 [6]. On the other hand, in a study conducted in Dhulikhel hospital, Nepal, 83.92% patients presented with VA of better than 6/12 and only 2.67% patients were under blindness category [8]. This might be due to discrepancies on the severity of ocular trauma.

There were variables missed in the chart and that cannot be presented and discussed here. Prospective study is recommended to address the missed variables in this study.

## Conclusion

The ocular trauma was more common in males and children. Majority of patients (98%) presented after 6 hours from time of ocular trauma and the treatment was commenced also after 6 hours for>95% of them. Cornea was the most commonly affected ocular structure by trauma. Ocular surgery, secondary to trauma, was performed in 53.17% patients and corneal tear repair was the most frequently performed procedure (51.8%).
